# The Semiflexible Polymer Translocation into Laterally Unbounded Region between Two Parallel Flat Membranes

**DOI:** 10.3390/polym8090332

**Published:** 2016-09-07

**Authors:** Zhi-Yong Yang, Ai-Hua Chai, Yong-Fu Yang, Xiao-Mao Li, Ping Li, Run-Ying Dai

**Affiliations:** 1 Department of Physics, Jiangxi Agricultural University, Nanchang 330045, China; zhiyongyang@163.com (Z.-Y.Y.); chinayangyf@163.com (Y.-F.Y.); lxmjxau@126.com (X.-M.L.); liping_jxau@163.com (P.L.); 2 College of Mathematics Physics and Information Engineering, Jiaxing University, Jiaxing 314001, China; caiaiwa@126.com; 3 Department of Chemistry, Jiangxi Agricultural University, Nanchang 330045, China

**Keywords:** Monte Carlo method, semiflexible polymer, translocation, scaling law

## Abstract

Using the dynamic Monte Carlo method, we investigate dynamics of semiflexible polymer translocation through a nanopore into laterally unbounded region between two parallel flat membranes with separation *R* in presence of an electric field inside the pore. The average translocation time τ initially decreases rapidly with increase of *R* in the range of *R* < 10 and then almost keeps constant for *R* ≥ 10, and the decline range increases with increase of dimensionless bending stiffness κ. We mainly study the effect of chain length *N*, κ and electric field strength *E* on the translocation process for *R* = 5. The translocation dynamics is significantly altered in comparison to an unconfined environment. We find τ ~ *N*^α^, where the exponent α increases with increase of *E* for small κ. α initially increases slowly with increase of *E* and then keeps constant for moderate κ. α decreases with increase of *E* for large κ. However, α decreases with increase of κ under various *E*. In addition, we find τ ~ κ^β^. β decreases with increase of *N* under various *E*. These behaviors are interpreted in terms of the probability distribution of translocation time and the waiting time of an individual monomer segment passing through the pore during translocation.

## 1. Introduction

The transport of proteins and nucleic acids through a nanopore is of essential importance to life. Representative examples include DNA and RNA translocation across nuclear pores, protein transport through membrane channel, and virus injection [1]. In 1996, Kasianowicz et al. [2] established what today the standard experimental method for studying the translocation of a macromolecule through a nanopore is. Over the past two decades, there has been considerable progress in the development of techniques for detecting and monitoring single-molecule translocation events. Much of this work has been motivated by the promise of an efficient and accurate translocation-based method for rapid DNA sequencing [3,4,5,6]. Other potentially revolutionary technological applications include protein analysis, filtration of macromolecules [7], molecular sieves [8], and controlled drug delivery [9].

Due to its wide range of applications, polymer translocation has been hot subject in recent years. The dynamics of a polymer’s translocation through nanopore is a complex and challenging problem. It can be affected by many factors, such as the driving force in the nanopore, the sequence of the polymer, the electrostatic interactions, the concentration, the flow of the fluid, the geometry of channel, the constitution of the channel and so on. To understand the translocation behaviors and uncover the underlying physical mechanism, scientists have done lots of work on studying the polymer translocation. Much of this work has been summarized in several recent reviews [10,11,12,13]. One important type of polymer translocation involves movement of polymers into or out of confined spaces. Recent theoretical and computer simulation studies in this area have mainly focused on the effect of different confined environment, such as spherical or ellipsoidal cavities [14,15,16,17,18,19,20,21,22,23,24,25,26,27,28,29,30,31,32,33,34,35,36,37], or laterally unbounded spaces between flat walls [38,39,40,41]. Much of this work is motivated by the problems of viral DNA packaging and ejection, in which DNA is confined to a space with dimensions comparable to its persistence length. Kindt et al. [42] have studied the translocation of DNA into a confined space at a constant packaging rate, and found that the force resisting DNA packaging rate is initially small, and then increases sharply after 40% of chain is packaged. It indicates that the translocation dynamics is greatly changed due to the entropic resisting force induced by crowding effect of the partially translocated monomers. Therefore, the process of polymer translocation into confined space is more complex, compared with an unconfined space. Up to now, scientists have done a lot of research on the polymer translocation into closed confined space, However, very little attention is paid to the polymer translocation into unclosed confined space [39,40]. Only Luo et al. employ Langevin dynamics simulations to investigate the dynamics of flexible polymer translocation into laterally unbounded spaces between two flat walls based on two-dimensional and three-dimensional model, respectively [39,40]. In two dimensions, they observe a nonuniversal dependence of the average translocation time τ on the chain length *N* [39]. In three dimensions, the confined space leads to nonuniversal dependence of average translocation time τ as a function of the driving force [40]. However, in order to capture some realistic aspects of a DNA translocation through a nanopore into laterally unbounded spaces between two flat walls, the chain stiffness should be considered [43,44,45]. To this end, we investigate the process of a semiflexible polymer translocation through nanopores into laterally unbounded spaces between two infinite parallel flat membranes by the dynamic Monte Carlo simulations. We mainly study the effect of the electric field strength and bending stiffness on the translocation dynamics.

## 2. Model and Methods

Dynamic Monte Carlo (DMC) method [46] based on three-dimensional off-lattice model is employed to study the process of semiflexible polymer chain translocation through a nanopore into laterally unbounded spaces between two infinite parallel flat walls. The chain consists of *N* + 1 effective monomers (where *N* is the chain length), and the neighboring monomers are connected by the finitely extendable nonlinear elastic (FENE) potential [47].

(1) $$U_{FENE}=-k{r_{0}}^{2}\ln[1-{\left(\frac{l_{i}-l_{0}}{r_{0}}\right)}^{2}]$$

Here, *l*_i_ is the length of *i*-th effective bond, which can vary in the range of *l*_min_ < *l*_i_ < *l*_max_ with *l*_min_ = 0.4 and *l*_max_ = 1.0, and its preferred distance *l*_0_ is 0.7 (where *l*_max_ is chosen to be the unit of length). *r*_0_ = *l*_max_ − *l*_0_ = *l*_0_ *− l*_min_, and the spring constant *k* is set to 20 in the units of *k_B_T* (where *k_B_* is the Boltzmann constant and *T* is the thermodynamic temperature). *k_B_T* is chosen to be the unit of energy.

Volume exclusion for all non-bonded monomers is imposed via a Morse-type potential [47].

(2) $$U_{M}=\sum_{\left|i-j\right|>1}\varepsilon (\exp(-2\alpha (r_{ij}-r_{\min}))-2\exp(-\alpha (r_{ij}-r_{\min})))$$

where *r_ij_* is the distance between the *i*-th monomer and the *j*-th monomer, and α = 24, *r*_min_ = 0.8, and ε = 1 are selected. Owing to the large value of α, $U_{M}$ decays to zero very rapidly for *r_ij_* > *r*_min_, and is completely negligible for distances larger than unit length. The combination of FENE bonds with excluded volume interactions is beneficial to prevent unphysical crossing of the polymers.

The bending stiffness used to describe the stiffness of polymer chain is modeled by an angle potential between adjacent bonds [47].

(3) $$U_{b}=\kappa (1+\cos\theta )$$

where θ is the angle between two consecutive bonds, and κ is the bending stiffness. The chain rigidity can be adjusted by varying κ. In addition, κ is in the units of *k_B_T*.

**Figure 1:**
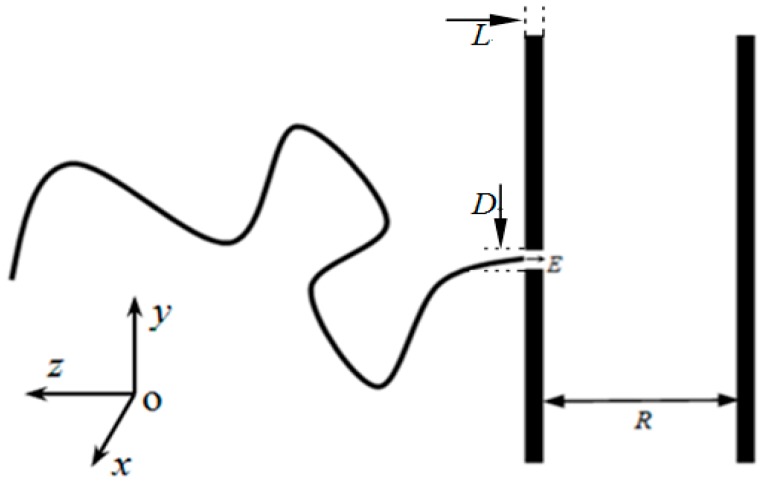
Schematic representation of polymer translocation into a confined space under the electric field strength *E* in the pore along −*z*-axis. The simulations are carried out in a planar confinement (3D), where two infinite parallel flat membranes are separated by a distance *R*. One membrane has a pore of length *L* = 2 and diameter *D* = 1.2.

We consider a geometry shown in Figure 1. The purely repulsive membranes consist of immobile monomers of diameter σ = *r*_min_. The Morse-type interaction between the immobile monomers of membrane and the mobile monomers of polymer is given by

(4) $$U_{W}=\{\begin{array}{lll} \varepsilon (\exp(-2\alpha (r-r_{\min}))-2\exp(-\alpha (r-r_{\min})))+\varepsilon & for & r\leq r_{\min}\\ 0 & for & r>r_{\min} \end{array}$$

where *r* is the distance between the immobile monomers of wall and the mobile monomers of polymer. In addition, there exists the same Morse-type interaction between the inside wall of the pore and the mobile monomers in the nanopore.

The two purely repulsive membranes are infinite, and the distance between the two purely repulsive membranes is *R*. The thickness of left membrane is *L*, and there is a nanopore of diameter *D* = 1.2 in it along −*z*-xis, as shown in Figure 1. Each monomer of polymer chain carries one effective charge *q* = 1, but the electrostatic force between monomers is neglected. Inside the pore, there exists a uniform electric field along the −*z*-axis, as shown in Figure 1. Once any monomer enters the pore, it will bear a repulsive force imposed by the inside wall of the pore and a uniform electric field force *f* = *qE* = *E*. The polymer is driven by the *f* to translocate along the −*z*-axis into laterally unbounded spaces between two flat membranes. If the monomer has a displacement −∆*z* along the *z*-axis, the electric field force will do work *A* = −∆*z*·*f* on the polymer. As we know, there is electric potential energy $U_{e}$ for the charged body in electric field. Therefore, the reduction of the polymer’s electric potential energy $\Delta U_{e}$ is equal to *A*.

Therefore, the total energy *U* of polymer can be written as:

(5) $$U=U_{FENE}+U_{M}+U_{b}+U_{W}+U_{e}$$

DMC simulations are performed, according to the Metropolis algorithm [48]. In more detail, For each trial move, a monomer is randomly selected and is attempted to move from its position (*x*_0_, *y*_0_, *z*_0_) to a new site (*x*, *y*, *z*) with increments ∆*x*, ∆*y* and ∆*z* which are chosen randomly from the intervals (−0.25, 0.25), respectively. The trial move is accepted, if ∆ > 1, where $\Delta =\min(\exp[-\Delta U/k_{B}T],1)$ is the transition probability depending on the difference in energy ΔU between the trial and old states. If ∆ = 1, a random function produces a random number η which is a number uniformly distributed in the interval [0, 1): if η > 0.5, the trial move is accepted, otherwise, the randomly selected monomer stays where it was. If ∆ < 1, random function produces a random number η: if ∆ > η, the trial move is accepted, otherwise, the randomly selected monomer stays where it was. The whole polymer chain can relax by repeating the above method. *N* + 1 trial moves are considered as one Monte Carlo step (MCS), which is chosen to be time unit.

Initially, the first monomer of the chain is fixed in the entrance of pore and the pore entrance is closed, while the remaining monomers are on the *cis* side. Then, the chain begins to relax on the *cis* side. Once the total energy of polymer chain fluctuates a little for a period of time, the polymer chain reaches equilibrium. The first monomer is released and the pore entrance opens, and this moment is set as time *t* = 0. The chain may withdraw from nanopore and drift away under the influence of the entropy barrier. If the chain withdraws from the nanopore, the translocation process restarts. Once the last monomer enters the *trans* side, the translocation process is over and the duration time is defined as translocation time. In order to avoid the high rate of withdrawing from the nanopore, the average acceptance rate of trial moves is in the range of (60%, 70%). The simulation results are averaged over 5000 independent samples. The run time changes with the model parameters. Here, we only provide the run time of a set of parameters. The run time of 5000 samples is about nine days on a computer cluster with the computer power of 399.36 teraflops for *E* = 5, κ = 40 and *N* = 150.

## 3. Results and Discussion

### 3.1. Effect of R on the Average Translocation Time

**Figure 2:**
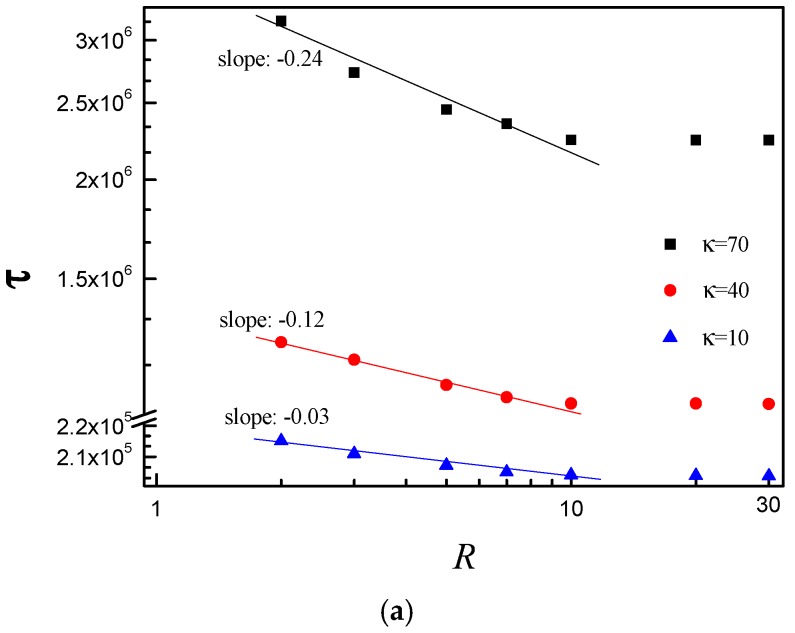

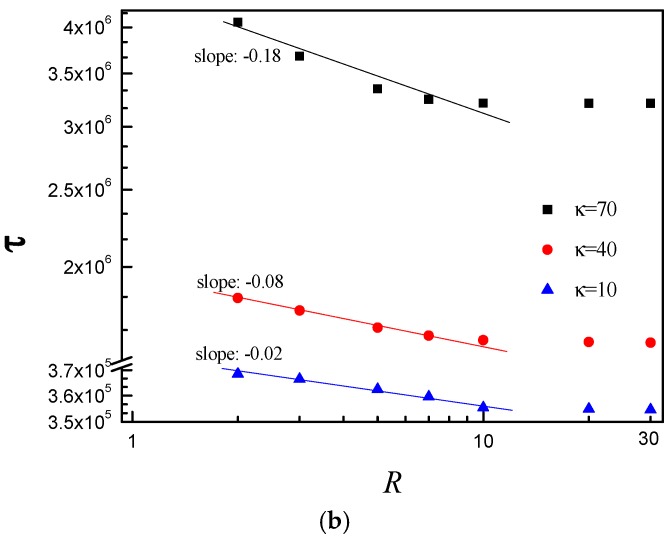
τ vs. the separation distance R between two flat membranes for: (**a**) *N* = 75, *E* = 2.5; and (**b**) *N* = 150, *E* = 5.0. Here, κ is the bending stiffness which can describe the stiffness of polymer chain. Data from *R* = 2 to 10 is plotted in log–log scale in Origin, and then the slope is obtained by the linear fit. The solid line is the linear fit of data from *R* = 2 to 10.

In this paper, we study the semiflexible polymer chain translocation through a nanopore into laterally unbounded space between two parallel flat membranes with separation *R*. We want to know if the *R* has effect on the translocation process. The Figure 2 shows the *R* dependence of the average translocation time τ for *E* = 2.5 and *N* = 75, *E* = 5.0 and *N* = 150, respectively. Figure 2a,b shows that the slope decreases with increase of κ in the range of *R* < 10, while τ almost keeps constant for *R* > 10. The results indicate that the translocation dynamics is influenced by confinement and the effect of confinement becomes more and more pronounced with increase of κ in the regime of *R* < 10.

### 3.2. Effect of E and b on Average Translocation Time

**Figure 3:**
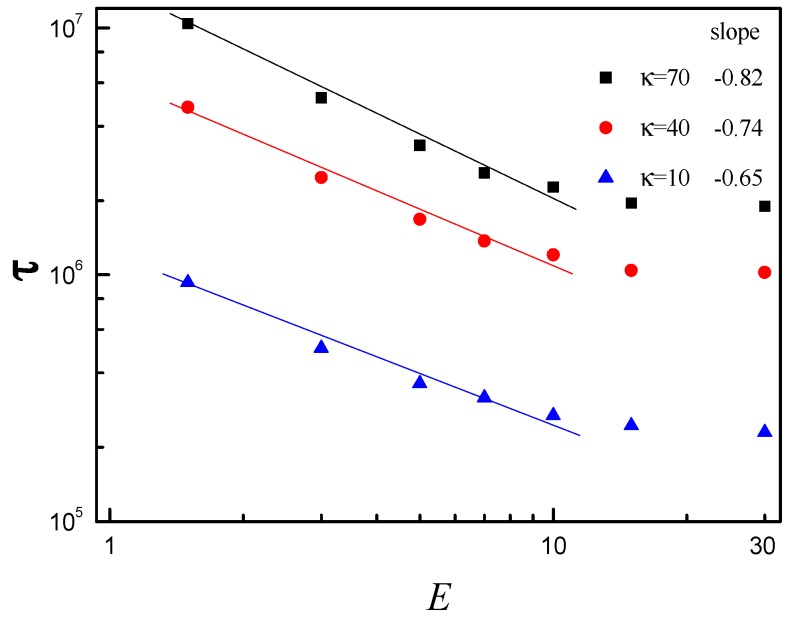
The average translocation time τ as a function of electric field strength *E* for bending stiffness κ = 10, 40 and 70. τ is obtained by averaging over the translocation times of 5000 samples. Data from *R* = 2 to 10 is plotted in log–log scale in Origin, and then the slope is obtained by the linear fit. The solid line is the linear fit of data from *R* = 2 to 10.

We examine the influence of the electric field strength *E* on the translocation dynamics by measuring the average translocation time τ. The *R* and *N* are set to *R* = 5 and *N* = 150, respectively. Figure 3 shows τ versus E in log–log scale plot for κ = 10, 40 and 70. It is found that the slope decreases from −0.65 to −0.82 with κ increasing from 10 to 70 in the range of 0 < *E* ≤ 10, i.e., the decline range of τ increases with increase of κ in the range of 0 < *E* ≤ 10. It indicates that *E* has more pronounced effect on the polymer translocation for larger κ. The main reason is that the interaction between the right membrane and the subchain on the *trans* side becomes more and more intense with increase of κ, and the interaction becomes much more intense with increase of *E*. τ almost keeps constant with increase of *E* in the range of *E* > 10 for κ = 10, 40 and 70. When the *E* is very large, confinement plays a dominant role in the translocation process. As a result, the effective driving force almost does not increase with increase of *E*.

### 3.3. Scaling Behavior of Average Translocation Time

**Figure 4:**
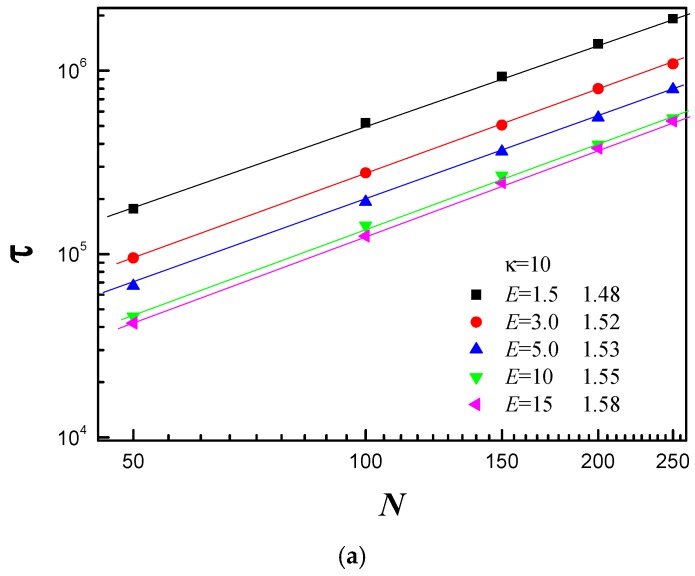

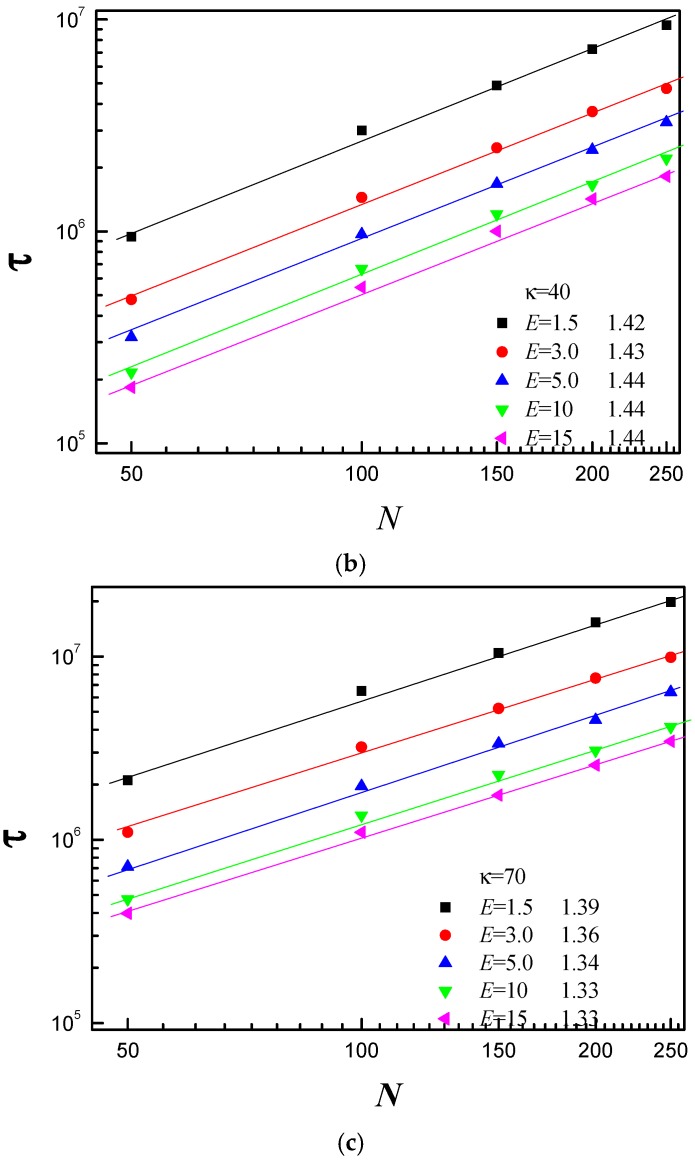
The average translocation time τ as a function of chain length *N* under the different electric field *E*: (**a**) for bending stiffness κ = 10; (**b**) for κ = 40; and (**c**) for κ = 70.

The chain length dependence of the average translocation time provides instructive information for better understanding of the specifics of the translocation process. Figure 4 shows τ as a function of the chain length *N* for different κ and *E*. Here, *R* is set to *R* = 5. We find τ ~ *N^α^* with α being the scaling exponent. Figure 4a shows the effect of *E* on the polymer’s scaling behavior for κ = 10. It can be observed that α increases from 1.48 to 1.58 with *E* increasing from 1.5 to 15. For κ = 10, the polymer chain is flexible. The electric field force has obvious effect on subchain conformation of the *cis* side. The partial chain close to the pore entrance first has to uncoil and become tense before moving into the pore according to tension propagation [13]. In addition, confinement produces pronounced effect on the subchain of the *trans* side. For flexible polymer chain, it is not easy for the translocated monomers to diffuse away from the nanopore exit. The confinement further makes translocated monomers harder to move away from the pore exit on the *trans* side. Therefore, there is crowding effect for κ = 10. With increase of *E*, the partial chain close to the pore entrance on the *cis* side becomes more and more tense, and the crowding effect becomes more and more pronounced on the *trans* side. This is the main reason why α increases with increase of *E*. Linna et al. also confirmed that the crowding effect leads to α’s increase with increase of external force [49]. Figure 4b shows the effect of *E* on the scaling behavior for κ = 40. We observe that α increases from 1.42 to 1.44 with increase of *E* in the range of *E* ≤ 5.0, then α keeps constant for larger *E*. For κ = 40, the polymer chain is of moderate stiffness. The subchain on the *cis* side is stretched. The tension propagation’s effect on the translocation dynamics becomes smaller. Its effect on the translocation process almost can be neglected. The conformation of subchain on the *trans* side is also stretched. If it touches the right flat membrane, it will interact with the right flat membrane. The interaction force is called as resisting force. The resisting force increases with increase of *E*. As a result, the effective driving force grows slowly with increase of *E*. Figure 4c shows that α decreases from 1.39 to 1.33 with *E* increasing from 1.5 to 15 for κ = 70. The polymer chain is stiff for κ = 70. The conformation is very stretched on the *cis* side before translocation. Therefore, there is no tension propagation on the *cis* side. The interaction between the subchain of the *trans* side and the right flat membrane is very intense. The resisting force drives the translocated subchain stretch itself in the *x*–*y* plane. In addition, the conformation of subchain on the *trans* side is very stretched. Therefore, there is no crowding effect for stiff chain.

**Figure 5:**
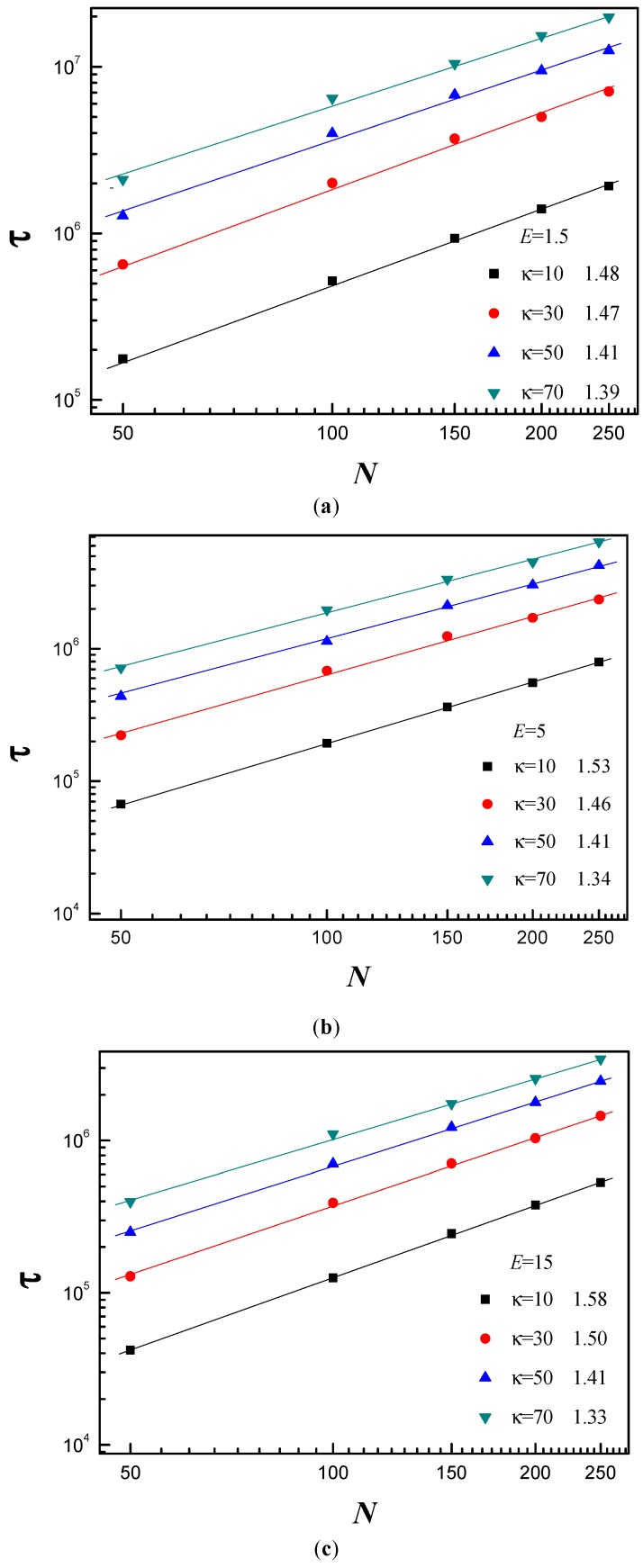
The average translocation time τ as a function of chain length *N* for the different κ at: (**a**) *E* = 1.5; (**b**) *E* = 5; and (**c**) *E* = 15.

Figure 5 shows that α decreases with increase of κ for *E* = 1.5, 5 and 15, and the decline range of α increases with increase of *E*. The tension propagation’s effect on the translocation process becomes smaller and smaller on the *cis* side with increase of κ. The free energy barrier increases with increase of κ. As a result, the polymer translocates more and more slowly with increase of κ. Therefore, the translocated monomers have enough time to diffuse away from the pore exit. In addition, the subchain becomes more and more stretched with increase of κ on the *trans* side. As a result, the crowding effect becomes weaker and weaker on the *trans* side with increase of κ, while the resisting force increases with κ. The driving force increases with increase of *E*. The interaction between the subchain of the *trans* side and right flat membrane becomes more and more intense with increase of *E*.

**Figure 6:**
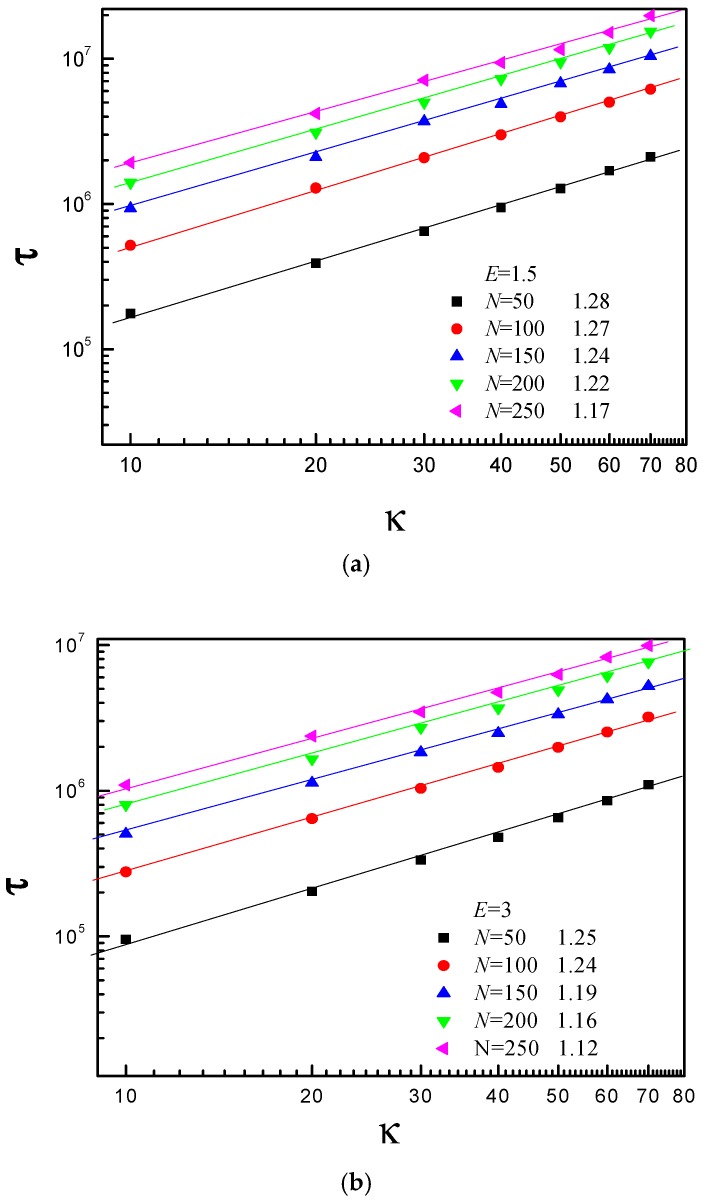

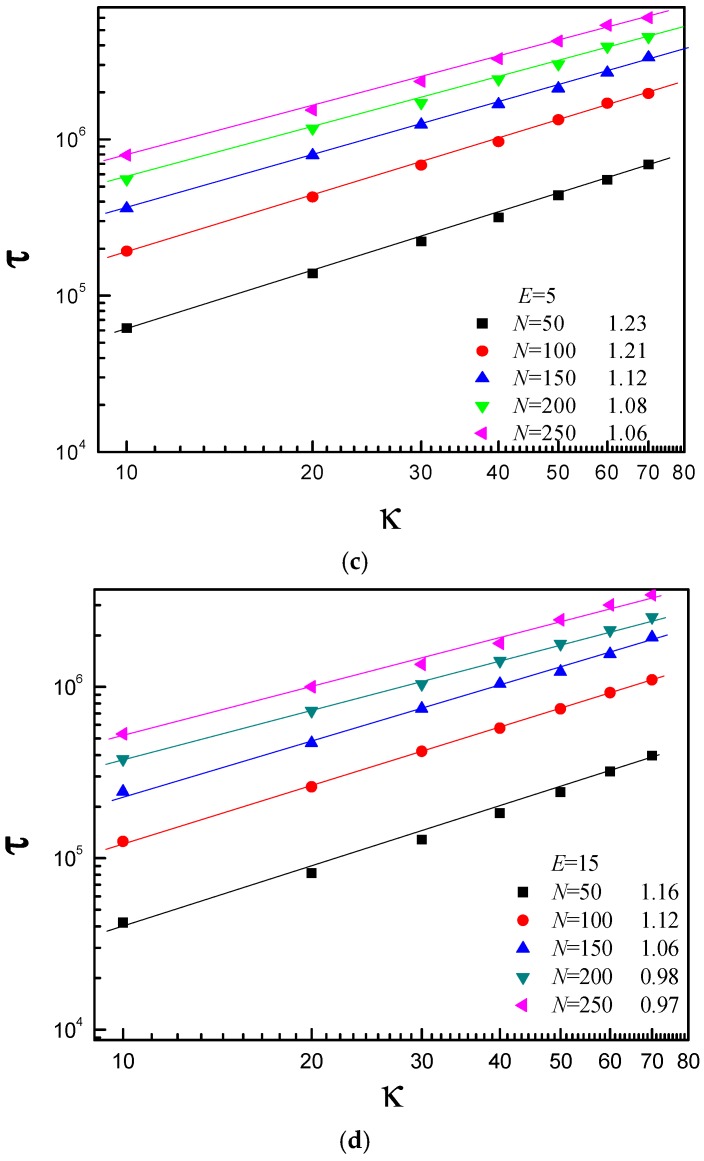
The average translocation time τ as a function of bending stiffness κ for the different chain length *N* at: (**a**) *E* = 1.5; (**b**) *E* = 3; (**c**) *E* = 5; and (**d**) *E* = 15. Here, *R* = 5.

To further understand the translocation process, the average translocation time τ as a function of bending stiffness κ is investigated for different *N*, as shown in Figure 6. We find that κ and τ also satisfy a scaling relation: τ ~ κ^β^, with β being the scaling exponent. Figure 6 shows that β decreases with increase of *N* for *E* = 1.5, 3, 5, and 15. For the short polymer chain, there is no crowding effect on the *trans* side. When the κ increases, the subchain of the *trans* side bears a resisting force, and it increases with increase of κ. For the long polymer chain, when κ is small, the crowding effect plays a dominant role in the translocation process, and there exists tension propagation on the *cis* side. When κ is large, effect of tension propagation becomes smaller and smaller with increase of κ. However, the interaction between the subchain of the *trans* side and the right membrane becomes more and more intense, i.e., the resisting force increases with increase of κ. In addition, the conformation of subchain on the *trans* side becomes more and more stretched with increase of κ. Therefore, crowding effect on the *trans* side becomes weaker and weaker. That is why the β decreases with increase of *N*. The free energy barrier decreases with increase of *E*, but the interaction between the subchain on the *trans* side and right flat membrane becomes more and more intense with increase of *E*, i.e., the resisting force increases with *E*. As a result, the effective driving force increases slowly. That is why the β decreases with increase of *E*.

**Figure 7:**
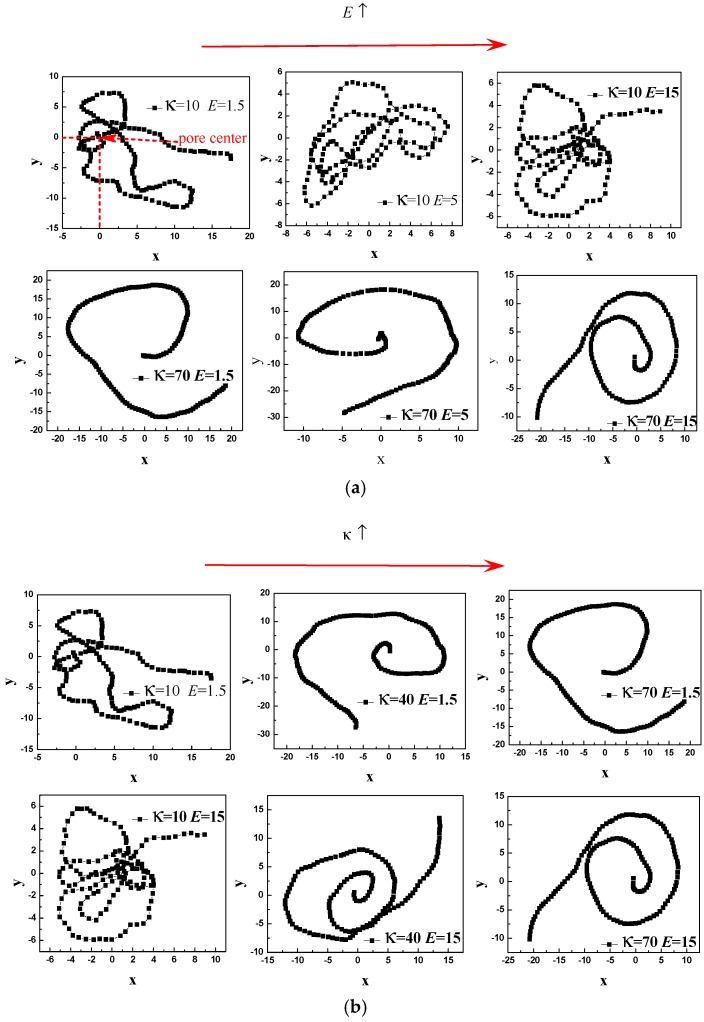
A three-dimensional polymer configuration right after translocation is projected onto the *x*–*y* plane for *N* = 150. The center of nanopore is at *x* = 0, *y* = 0. (**a**) The influence of *E* on the configuration; (**b**) The influence of κ on the configuration.

To give more detail, the Figure 7 shows the just translocated polymer’s configuration projected onto *x*–*y* plane. Figure 7a shows that the conformation of κ = 10 is coil, and it becomes more and more compact with increase of *E*. In addition, the monomer density around the pore exit increases with increase of *E* for κ = 10 (where the position of pore center projected onto *x*–*y* plane is *x* = 0 and *y* = 0). For κ = 70, we can observe toroidal structure, and the number of helix turn increases with increase of *E*. It verifies that the stiff polymers bears a resisting force on the *trans* side and the resisting force increases with increase of *E*. It can also be observed that the monomer density around the nanopore exit is very small. Figure 7b shows that the monomer density around the nanopore exit decreases with increase of κ for both *E* = 1.5 and 15. It can be observed that the configuration is toroidal for moderate and strong stiffness, and the number of helix turn decreases with increase of κ. It verifies that the resisting force increases with κ under any *E*. In addition, the monomer density around the nanopore exit is small for moderate and strong stiffness under any electric field.

### 3.4. Time Distribution

**Figure 8:**
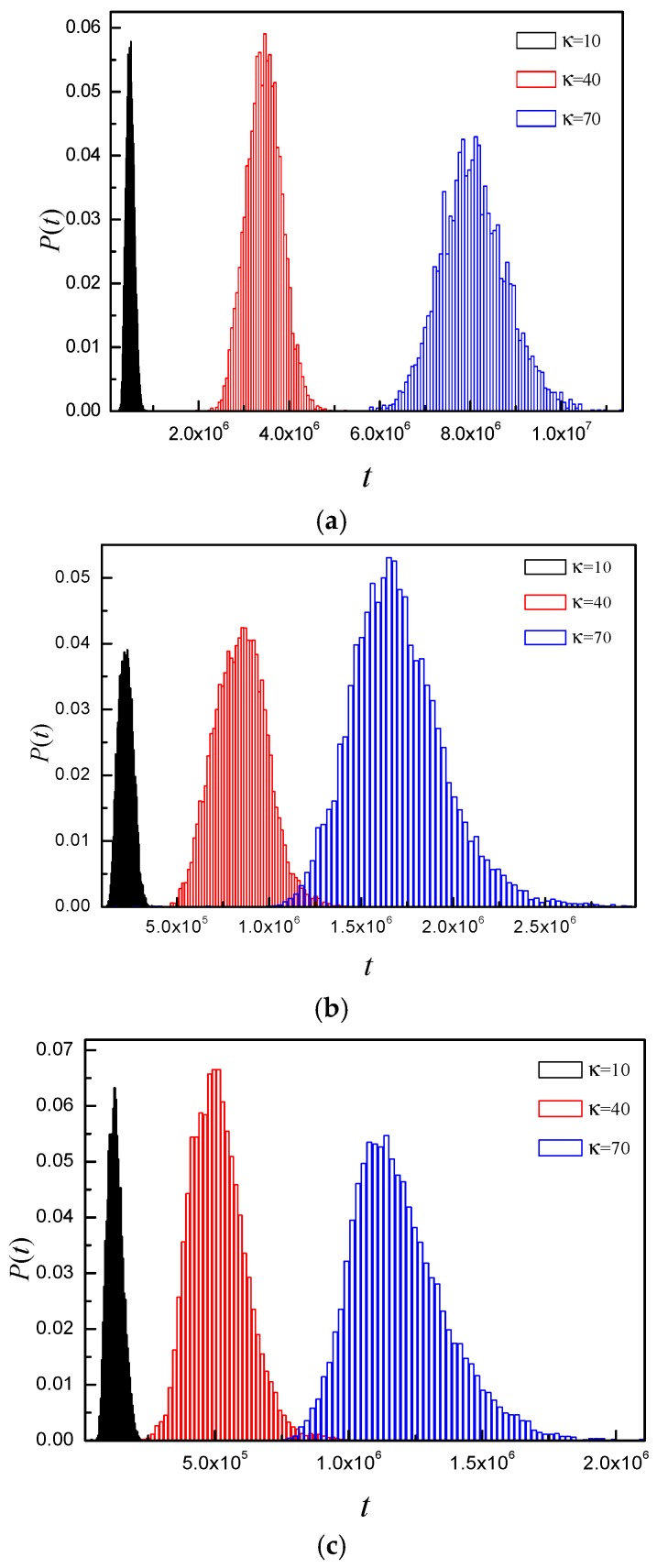
The probability distribution of the translocation time for different κ at: (**a**) *E* = 1.5; (**b**) *E* = 5; and (**c**) *E* = 15. Here, *R* = 5, *N* = 150.

Firstly, we study probability distribution of translocation time. It can tell us the detail of the whole translocation process. Here, the chain length is set to *N* = 150. Figure 8 shows the probability distribution of translocation times for different *E* and κ. Figure 8a shows that the histograms of κ = 10, 40, and 70 take on Gaussian distributions under *E* = 1.5, but the position of the peak shifts to a higher value and the distribution becomes broader and broader with increase of κ. The strength of electric field is so small that the electric field force cannot overcome the free energy barrier. The polymer chain translocate through the nanopore by biased diffusion. The free energy barrier becomes larger and larger and the resisting force increases with increase of κ. Therefore, the polymer translocates more and more slowly with increase of κ. As a result, there is no crowding effect on the *trans* side. Figure 8b shows distribution of translocation time for different κ at *E* = 5. The histogram of κ = 10 takes on a nearly Gaussian distribution. The electric field force is large enough to overcome the free energy barrier. The polymer chain can translocate quickly, however, there is crowding effect on the *trans* side. For κ = 40 and 70, an asymmetric distribution with a right tail is observed. The larger κ is, the longer the tail is. The free energy barrier increases with κ. In addition, the resisting force increases with increase of κ. As a result, the effective driving force decreases with increase of κ. Figure 8c shows asymmetric distribution with a right tail for κ = 10, 40, and 70 under *E* = 15. However, the distribution width increases, and the tail becomes longer and longer with increase of κ. The *E* is large enough to ensure that the polymer translocates quickly. For κ = 10, the crowding effect becomes more obvious at *E* = 15, and it plays dominant role in the translocation process. For large κ, interaction between the subchain of the *trans* side and the right membrane becomes more intense at *E* = 15, and the interaction becomes more and more intense with increase of κ.

**Figure 9:**
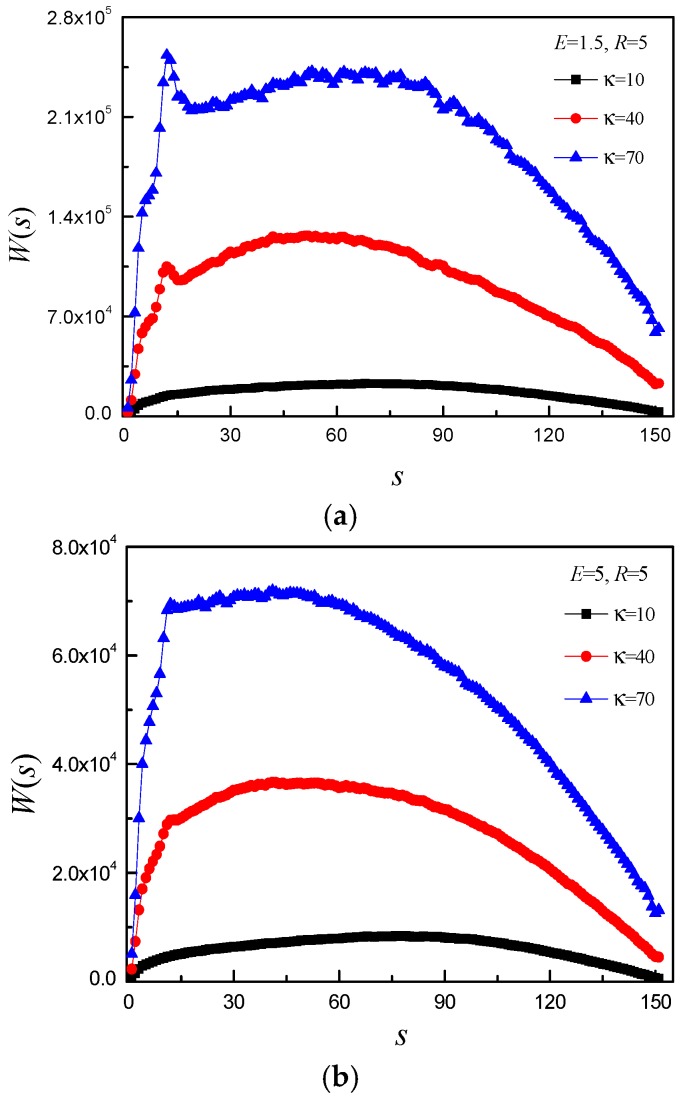

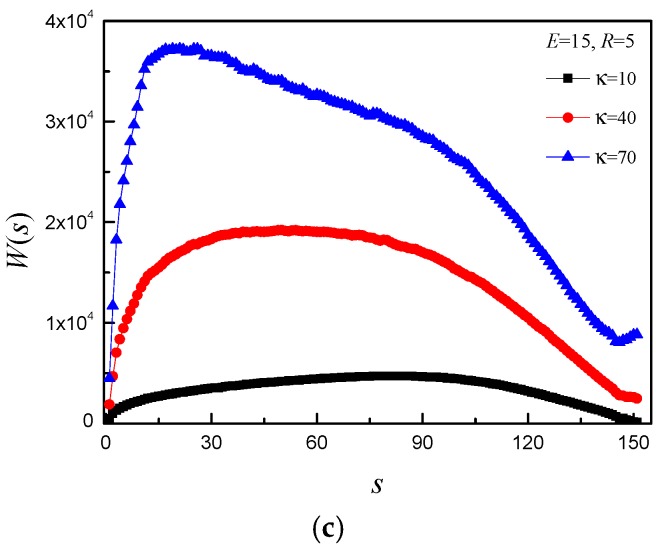
The waiting time distribution *W*(*s*) as a function of monomer *s* for *N* = 150 and different κ under: (**a**) *E* = 1.5; (**b**) *E* = 5; and (**c**) *E* = 15. Here, *R* = 5.

Next, to further understand the translocation mechanisms, which are considerably affected by the non-equilibrium nature of the translocation process, we explore the dynamics of a single monomer passing through the nanopore into confined space. We numerically calculate the waiting time of the monomer *s*, which is defined as the amount of time which monomer *s* spends inside the pore and is averaged over the different simulation trajectories. Evidently, a plot of *W*(*s*) as a function of *s* reveals detailed information about the translocation process of the individual monomer. This quantity has been studied in detail in the past for fully flexible chains and more recently for semiflexible chains. Here, *N* and *R* are set to 150 and 5, respectively. Figure 9 shows *W*(*s*) for different κ and *E*. It is easily observed that the *W*(*s*) can be divided into two stages: the initial stage of increasing *W*(*s*) and the second stage of decreasing *W*(*s*), and the *E* has obvious effect on the *W*(*s*) of κ = 40 and 70. Figure 9a shows that *W*(*s*) of κ = 40 and 70 is very different from *W*(*s*) of κ = 10. For κ = 40 and 70, the *W*(*s*) increases sharply before *s* = 12 and a small peak appears at *s* = 12 in the initial stage of *W*(*s*). The peak becomes steeper and steeper with increase of κ. If the subchain of the *trans* side just touches the right flat membrane, it will bear resisting force for κ = 40 and 70 and the resisting force increases with time until it drives the subchain stretch itself in the *x*–*y* plane. The peak indicates that the subchain on the *trans* side begins to stretch itself in the *x*–*y* plane. At *E* = 5, the only change of κ = 10 is that the maximum of *W*(*s*) shifts to a higher *s*-value. The *W*(*s*) still increases sharply before *s* = 12 for κ = 40 and 70, then it increases very slowly, even almost keeps constant for κ = 70 in the initial stage of *W*(*s*), as shown in Figure 9b. At *E* = 15, the maximum of *W*(*s*) shifts to a lower *s*-value with increase of κ, as shown in Figure 9c. For κ = 10, the waiting time distribution still does not have obvious change, except the maximum of *W*(*s*) shifting to a much higher *s*-value. For κ = 40, the *W*(*s*) increases with *s*, then almost keeps constant with increase of *s* for the middle part of monomers, Finally decreases with increase of *s*. For κ = 70, the *W*(*s*) increases quickly to maximum with *s*, then the decreasing stage can be divided into two processes: one is the slowly decreasing process, another is the quickly decreasing process. For κ = 10, the maximum of *W*(*s*) shifts to a higher *s*-value with increase of *E*. It indicates that there is crowding effect under large *E*. The *W*(*s*) of κ = 40 is different from that of κ = 70 under different *E*. It implies that their translocation dynamics is different.

**Figure 10:**
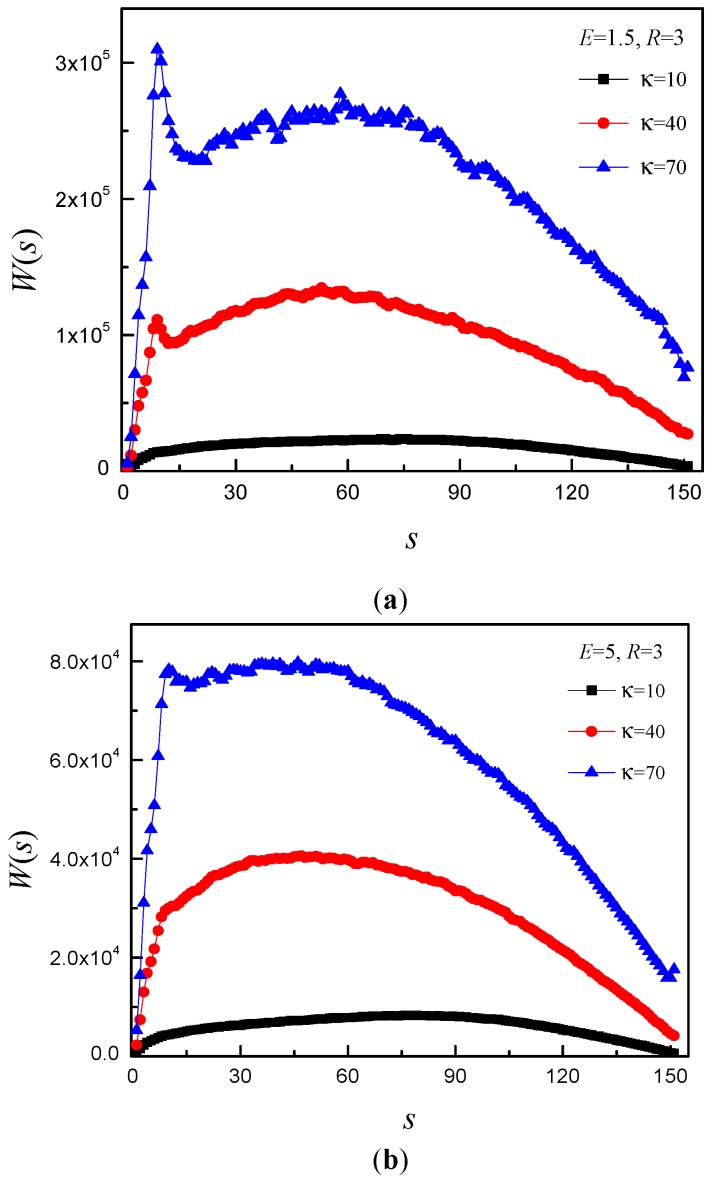

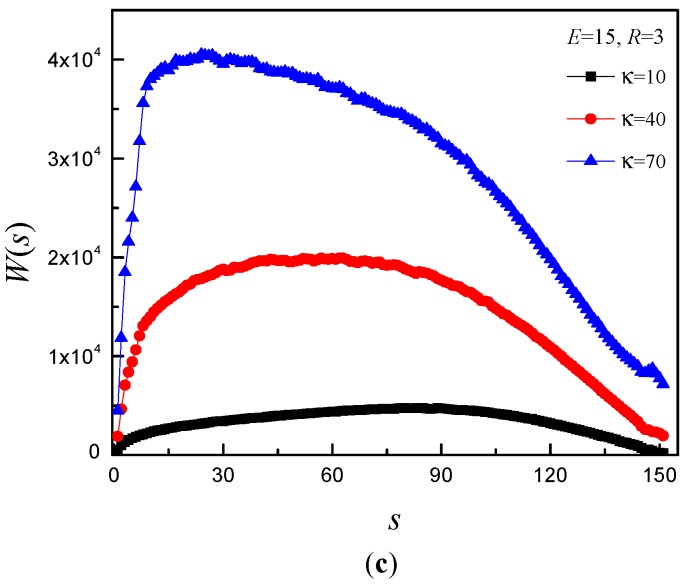
The waiting time distribution *W*(*s*) as a function of monomer *s* for *N* = 150 and different κ under: (**a**) *E* = 1.5; (**b**) *E* = 5; and (**c**) *E* = 15. Here, *R* = 3.

Figure 10 shows that the *W*(*s*) has the same changing trend for *R* = 3, however, the position of peak or cusp shifts to *s* = 9. It indicates that the *R* has effect on the position of peak or cusp. The position of peak indicates that the subchain of the *trans* side just touches the right flat membrane. In order to let the monomers translocate into the *trans* side, the subchain must stretch itself in *x*–*y* plane. When *E* is small, the resisting force is so small that the subchain on the *trans* side needs lots of time to adjust its position to bend itself and stretch itself in *x*–*y* plane. The larger κ is, the more rigid the chain is. Therefore, the adjustment time is longer for larger κ. That is why there is a peak for large κ, and the peak becomes steeper and steeper with increase of κ at small *E*. When *E* is large, the resisting force is so large that subchain of the *trans* side does not need to adjust its position to bend and extend itself in *x*–*y* plane. That is why there is cusp for large κ and *E.*

## 4. Conclusions

We have studied translocation of semiflexible polymers through a nanopore into laterally unbounded space between two infinite parallel flat membranes with separation *R* in 3D, using dynamic Monte Carlo method. The confinement produces obvious effect on the translocation process when the *R* is less than 10. We investigate the dynamics of semiflexible polymer moving into the region between two infinite parallel membrane with *R* = 5 in detail. We mainly study the effect of bending stiffness κ and electric field strength *E* on the translocation process. We find τ ~ *N*^α^. For weak stiffness, α increases with increase of *E*. For moderate stiffness, α firstly increases, then keeps constant with increase of *E*. For strong stiffness, α decreases with increase of *E*. It can be concluded that their translocation dynamics is different for polymers of different rigidity. In addition, we find τ ~ κ^β^. β decreases with increase of *N* under various *E*. The results of a three-dimensional polymer configuration right after translocation projected onto the *x*–*y* plane show that there is crowding effect in the translocation process for small stiffness; the conformation is toroidal for moderate and strong stiffness, and the number of helix turns increases with κ or *E*. It indicates that the subchain on the *trans* side bears resisting force which is imposed by the right flat membrane, and the resisting force increases with κ and *E*. It can be used to interpret the scaling behavior. In order to further understand the translocation process, we study the probability distribution of translocation time and waiting time distribution at various κ and *E*. They can further interpret why the κ and *E* have effect on the translocation process.
